# Supplementary material for: Comprehensive Comparative Analysis Sheds Light on the Patterns of Microsatellite Distribution across Birds Based on the Chromosome-Level Genomes
Source: Animals (Basel). 2023 Feb 13;13(4):655. doi: 10.3390/ani13040655 (PMC9951716; doi:10.3390/ani13040655)
Supplement: Supplementary file 1 [file animals-13-00655-s001.zip › animals-2052787-supplementary.pdf]

SUPPLEMENTARY MATERIALS

Table S1. Species information of SSRs analysis.

Order	Species	Assembly level	Genome coverage	Sequencing technology	Genome size (bp)	Accession
Galliformes	*Coturnix japonica*	Chromosome	73x	Illumina GA IIx	927656957	GCF_001577835.2
Galliformes	*Meleagris gallopavo*	Chromosome	35.0x	Illumina GAII; Sanger; 454	1115474681	GCF_000146605.3
Galliformes	*Gallus gallus*	Chromosome	102.01x	PacBio Sequel I CLR; Illumina NovaSeq; Arima Genomics Hi-C; Bionano Genomics DLS	1053332251	GCF_016699485.2
Galliformes	*Numida meleagris*	Chromosome	100.0x	Illumina HiSeq	1043264150	GCF_002078875.1
Anseriformes	*Oxyura jamaicensis*	Chromosome	60.0x	Illumina NovaSeq	1147585764	GCF_011077185.1
Anseriformes	*Heteronetta atricapilla*	Chromosome	60.0x	Illumina NovaSeq	1151880818	GCA_011075105.1
Anseriformes	*Cygnus olor*	Chromosome	60.23x	PacBio Sequel I CLR; Illumina NovaSeq; Arima Genomics Hi-C; Bionano Genomics DLS	1134186642	GCF_009769625.2
Anseriformes	*Aythya fuligula*	Chromosome	64.03x	PacBio Sequel I CLR; Illumina NovaSeq; Arima Genomics Hi-C; Bionano Genomics DLS	1127004725	GCF_009819795.1
Anseriformes	*Anas platyrhynchos*	Chromosome	143.0x	PacBio RSII; PacBio Sequel	1188533289	GCF_015476345.1
Anseriformes	*Stictonetta naevosa*	Chromosome	60.0x	Illumina NovaSeq	1132698962	GCA_011074415.1
Anseriformes	*Cairina moschata*	Chromosome	128.0x	Illumina NovaSeq	1118556028	GCA_018104995.1
Piciformes	*Colaptes auratus*	Chromosome	34.0x	Illumina NovaSeq; PacBio Sequel	1378187165	GCA_015227895.1
Piciformes	*Pogoniulus pusillus*	Chromosome	165.44x	PacBio Sequel I CLR; Illumina NovaSeq; Arima Genomics Hi-C; Bionano Genomics DLS	1272358903	GCA_015220805.1
Bucerotiformes	*Bucorvus abyssinicus*	Chromosome	66.0x	PacBio Sequel I CLR; Illumina NovaSeq; Arima Genomics Hi-C; Bionano Genomics DLS	1132597561	GCA_009769605.1

Psittaciformes	*Myiopsitta monachus*	Chromosome	67.0x	PacBio RSII	1168583304	GCA_017639245.1
Psittaciformes	*Amazona aestiva*	Chromosome	60.0x	Illumina	1130393257	GCA_017639355.1
Psittaciformes	*Melopsittacus undulatus*	Chromosome	61.11x	PacBio Sequel I CLR; Illumina NovaSeq; Arima Genomics Hi-C; Bionano Genomics DLS	1171617451	GCF_012275295.1
Passeriformes	*Rhegmatorhina hoffmannsi*	Chromosome	141.0x	Illumina HiSeq	1066555733	GCA_013398505.2
Passeriformes	*Certhia americana*	Chromosome	80.0x	PacBio Sequel; Illumina HiSeq; Illumina NovaSeq	1113356413	GCA_018697195.1
Passeriformes	*Hirundo rustica*	Chromosome	51.15x	PacBio Sequel I CLR; Illumina NovaSeq; Arima Genomics Hi-C; Bionano Genomics DLS	1105955550	GCF_015227805.1
Passeriformes	*Catharus ustulatus*	Chromosome	60.58x	PacBio Sequel I CLR; Illumina NovaSeq; Arima Genomics Hi-C; Bionano Genomics DLS	1131616530	GCF_009819885.2
Passeriformes	*Erithacus rubecula*	Chromosome	41x	PacBio Sequel I CLR; Illumina NovaSeq; Arima Genomics Hi-C; Bionano Genomics DLS	1086738418	GCA_903797595.2
Passeriformes	*Ficedula albicollis*	Chromosome	60.0x	Illumina GAII; Illumina HiSeq	1118343587	GCF_000247815.1
Passeriformes	*Sylvia atricapilla*	Chromosome	66.29x	PacBio Sequel I CLR; Illumina NovaSeq; Arima Genomics Hi-C; Bionano Genomics DLS	1066786587	GCA_009819655.1
Passeriformes	*Sylvia borin*	Chromosome	72.07x	PacBio Sequel I CLR; Illumina NovaSeq; Arima Genomics Hi-C; Bionano Genomics DLS	1045652180	GCA_014839755.1
Passeriformes	*Acrocephalus scirpaceus*	Chromosome	200x	Unknown	1075083815	GCA_910950805.1
Passeriformes	*Parus major*	Chromosome	95.0x	Illumina HiSeq	1020310769	GCF_001522545.3
Passeriformes	*Camarhynchus parvulus*	Chromosome	31x	Unknown	1051609828	GCF_901933205.1
Passeriformes	*Diglossa brunneiventris*	Chromosome	30.0x	PacBio Sequel	1079473785	GCA_019023105.1
Passeriformes	*Passer domesticus*	Chromosome	130.0x	Illumina HiSeq	1042720703	GCA_001700915.1
Passeriformes	*Molothrus ater*	Chromosome	200.0x	PacBio Sequel II	1087312585	GCF_012460135.1

Passeriformes	*Setophaga coronata*	Chromosome	100.0x	Illumina	1022651334	GCA_001746935.2
Passeriformes	*Geothlypis trichas*	Chromosome	52.11x	PacBio Sequel I CLR; Illumina NovaSeq; Arima Genomics Hi-C; Bionano Genomics DLS	1078128490	GCA_009764595.1
Passeriformes	*Motacilla alba*	Chromosome	18x	PacBio Sequel II HiFi	1072670728	GCF_015832195.1
Passeriformes	*Fringilla coelebs*	Chromosome	249.0x	Illumina HiSeq	994865760	GCA_015532645.2
Passeriformes	*Lonchura striata*	Chromosome	151.0x	Illumina HiSeq	1060269806	GCF_005870125.1
Passeriformes	*Taeniopygia guttata*	Chromosome	Unknown	PacBio	1056271262	GCF_003957565.2
Passeriformes	*Corvus moneduloides*	Chromosome	62.08x	PacBio Sequel I; Illumina NovaSeq; Arima Genomics Hi-C; Bionano Genomics DLS	1112734094	GCF_009650955.1
Passeriformes	*Corvus monedula*	Chromosome	64.0x	PacBio; Hi-C	1035863955	GCA_013407035.1
Passeriformes	*Malurus cyaneus*	Chromosome	51.2x	PacBio RSII; Illumina HiSeq; Illumina MiSeq	1078891882	GCA_009741485.1
Columbiformes	*Streptopelia turtur*	Chromosome	44x	PacBio Sequel I; Illumina NovaSeq; Arima Genomics Hi-C; Bionano Genomics DLS	1178816852	GCA_901699155.2
Phoenicopteriformes	*Phoenicopterus ruber*	Chromosome	61.27x	PacBio Sequel I CLR; Illumina NovaSeq; Arima Genomics Hi-C; Bionano Genomics DLS	1246226932	GCA_009819775.1
Ciconiiformes	*Ciconia maguari*	Chromosome	88.32x	PacBio Sequel II CLR; Illumina NovaSeq; Arima Genomics Hi-C; Bionano Genomics DLS	1239437416	GCA_017639555.1
Cathartiformes	*Gymnogyps californianus*	Chromosome	20.0x	PacBio RSII; Phase Genomics HiC	1240179868	GCA_018139145.1
Charadriiformes	*Alca torda*	Chromosome	64.69x	PacBio Sequel; Illumina NovaSeq; 10X Genome; Arima Hi-C	1178492568	GCA_008658365.1
Charadriiformes	*Sterna hirundo*	Chromosome	67.91x	PacBio Sequel I CLR; Illumina NovaSeq; Arima Genomics Hi-C; Bionano Genomics DLS	1229972541	GCA_009819605.1
Charadriiformes	*Pluvialis apricaria*	Chromosome	60.0x	PacBio Sequel II CLR; Illumina NovaSeq; Arima Genomics Hi-C; Bionano Genomics DLS	1247767512	GCA_017639485.1

Falconiformes	*Falco naumanni*	Chromosome	104.34x	PacBio Sequel I CLR; Illumina NovaSeq; Arima Genomics Hi-C; Bionano Genomics DLS	1215719661	GCF_017639655.2
Falconiformes	*Falco rusticolus*	Chromosome	44.28x	PacBio Sequel I CLR; Illumina NovaSeq; Arima Genomics Hi-C; Bionano Genomics DLS	1195847496	GCF_015220075.1
Accipitriformes	*Aquila chrysaetos*	Chromosome	60x	PacBio Sequel I CLR; Illumina NovaSeq; Arima Genomics Hi-C; Bionano Genomics DLS	1233704830	GCF_900496995.4
Caprimulgiformes	*Calypte anna*	Chromosome	54.0x	PacBio RSII; 10X Genomics linked reads; Bionano Genomics DLS; Arima Genomics HiC	1059687259	GCF_003957555.1
Caprimulgiformes	*Caprimulgus europaeus*	Chromosome	87x	PacBio	1177791212	GCA_907165065.1
Caprimulgiformes	*Nyctibius grandis*	Chromosome	88.4x	PacBio Sequel I CLR; Illumina NovaSeq; Arima Genomics Hi-C; Bionano Genomics DLS	1256414880	GCA_013368605.1

Table S2. The number of different SSR types.

Species	Number of three SSR types				Number of six P-SSR types					
	Total	C-SSR	I-SSR	P-SSR	Mono-	Di-	Tri-	Tetra-	Penta-	Hexa-
Coturnix japonica	1206213	18271	925257	262685	133890	30357	23844	50784	19476	4334
Meleagris gallopavo	1265444	7413	1022305	235726	138861	25204	19857	36605	12414	2785
Gallus gallus	1411711	15856	1081648	314207	192740	27640	24452	42210	21404	5761
Numida meleagris	1199459	6884	967765	224810	161606	16696	14988	20530	8881	2109
Oxyura jamaicensis	2005910	34605	1413610	557695	349210	46121	52444	76181	29319	4420
Heteronetta atricapilla	1961030	30645	1404201	526184	331094	46307	49410	70241	25108	4024
Cygnus olor	1854591	27228	1351045	476318	291568	38832	40694	61821	32743	10660
Aythya fuligula	2080570	40930	1454294	585346	367470	44730	45811	81765	35363	10207
Anas platyrhynchos	2068309	36550	1482880	548879	327692	44738	43836	80680	39084	12849
Stictonetta naevosa	1875358	25268	1359084	491006	306880	36739	44960	70680	26971	4776
Cairina moschata	2004732	31704	1426106	546922	351659	44295	41955	75178	27824	6011
Colaptes auratus	1630446	28920	1286013	315513	103043	30357	23014	36788	116021	6290
Pogoniulus pusillus	1273033	11525	1061581	199927	94906	29543	27126	29414	15244	3694
Bucorvus abyssinicus	1197660	5689	968972	222999	163378	14979	17369	14691	10478	2104

Myiopsitta monachus	1193100	7715	986814	198571	129866	16947	15813	24443	9186	2316
Amazona aestiva	935817	5118	781069	149630	104108	13148	10223	15460	5414	1277
Melopsittacus undulatus	1124634	5151	951607	167876	102848	17467	13077	24928	6407	3149
Rhegmatorhina hoffmannsi	1037755	6599	876901	154255	76584	21869	17879	23508	10650	3765
Certhia americana	1311143	15572	1012166	283405	185434	28171	22069	28241	14097	5393
Hirundo rustica	1453654	24673	1075224	353757	230875	27580	26377	40476	22005	6444
Catharus ustulatus	1248256	12804	1017876	217576	116588	18353	22001	34139	18999	7496
Erithacus rubecula	1341694	18682	1033586	289426	172994	24023	21178	40120	23403	7708
Ficedula albicollis	1394779	31267	1042916	320596	216726	21308	18832	38410	16383	8937
Sylvia atricapilla	1277875	16507	990662	270706	178464	22582	22676	25340	17182	4462
Sylvia borin	1238537	14941	975681	247915	158854	21280	23389	25044	14275	5073
Acrocephalus scirpaceus	1162762	10900	946581	205281	118625	19668	21710	25991	14844	4443
Parus major	1086323	7621	900149	178553	100191	23808	20132	19460	10283	4679
Camarhynchus parvulus	1136783	12917	933498	190368	88348	21559	23694	30668	20656	5443
Diglossa brunneiventris	1221936	13968	981917	226051	123787	23703	22361	31991	19635	4574
Passer domesticus	1030645	5644	861297	163704	99506	16270	16408	19055	9721	2744
Molothrus ater	1238753	11787	1005981	220985	115606	27267	26520	30313	16413	4866

Setophaga coronata	948979	4816	802844	141319	82000	17907	15420	16384	7876	1732
Geothlypis trichas	1165658	13231	952893	199534	98540	25671	22025	30797	18631	3870
Motacilla alba	1200479	12083	960720	227676	136111	23725	21455	28214	13701	4470
Fringilla coelebs	1044105	5637	868571	169897	105464	16556	17704	18337	8953	2883
Lonchura striata	1190217	17267	940324	232626	149392	21006	19996	22166	13548	6518
Taeniopygia guttata	1247305	15203	975477	256625	160950	20478	20474	26615	21423	6685
Corvus moneduloides	1122068	8963	918233	194872	113661	19692	19095	25287	11997	5140
Corvus monedula	1051173	9068	856330	185775	111017	17366	16974	24518	12754	3146
Malurus cyaneus	1276017	16537	1007121	252359	168175	17552	16935	26202	19047	4448
Streptopelia turtur	1358590	12101	1073064	273425	177511	24261	17841	34784	15960	3068
Phoenicopterus ruber	1376453	7374	1111063	258016	187532	16258	21178	19075	11716	2257
Ciconia maguari	1327554	7439	1084256	235859	162002	18250	22010	17654	12823	3120
Gymnogyps californianus	1428162	7276	1149500	271386	190967	17281	29662	19457	11485	2534
Alca torda	1503572	12074	1171425	320073	243793	20962	17500	22382	12933	2503
Sterna hirundo	1509488	10890	1187831	310767	234262	20872	20140	21328	11602	2563
Pluvialis apricaria	1455786	9294	1166013	280479	207134	18561	20269	18722	12356	3437
Falco naumanni	1431181	11570	1141991	277620	202431	23592	19152	21244	8948	2253

Falco rusticolus	1378322	9449	1111283	257590	185864	23100	18600	19669	8510	1847
Aquila chrysaetos	1512190	9854	1180798	321538	245911	20247	23749	19969	9859	1803
Calypte anna	1355939	13766	1073222	268951	173511	24237	19014	28458	18506	5225
Caprimulgus europaeus	1438778	11478	1161094	266206	169093	28206	22800	26490	15109	4508
Nyctibius grandis	1288597	4973	1082013	201611	144754	16074	16916	13259	8119	2489

Table S3. The frequency of different SSR types.

Species	Frequency of three SSR types			Frequency of six P-SSR types					
	C-SSR (%)	I-SSR (%)	P-SSR (%)	Mono- (%)	Di- (%)	Tri- (%)	Tetra- (%)	Penta- (%)	Hexa- (%)
Coturnix japonica	1.51	76.71	21.78	50.97	11.56	9.08	19.33	7.41	1.65
Meleagris gallopavo	0.59	80.79	18.63	58.91	10.69	8.42	15.53	5.27	1.18
Gallus gallus	1.12	76.62	22.26	61.34	8.80	7.78	13.43	6.81	1.83
Numida meleagris	0.57	80.68	18.74	71.89	7.43	6.67	9.13	3.95	0.94
Oxyura jamaicensis	1.73	70.47	27.80	62.62	8.27	9.40	13.66	5.26	0.79
Heteronetta atricapilla	1.56	71.61	26.83	62.92	8.80	9.39	13.35	4.77	0.76
Cygnus olor	1.47	72.85	25.68	61.21	8.15	8.54	12.98	6.87	2.24
Aythya fuligula	1.97	69.90	28.13	62.78	7.64	7.83	13.97	6.04	1.74
Anas platyrhynchos	1.77	71.70	26.54	59.70	8.15	7.99	14.70	7.12	2.34
Stictonetta naevosa	1.35	72.47	26.18	62.50	7.48	9.16	14.39	5.49	0.97
Cairina moschata	1.58	71.14	27.28	64.30	8.10	7.67	13.75	5.09	1.10
Colaptes auratus	1.77	78.87	19.35	32.66	9.62	7.29	11.66	36.77	1.99
Pogoniulus pusillus	0.91	83.39	15.70	47.47	14.78	13.57	14.71	7.62	1.85
Bucorvus abyssinicus	0.48	80.91	18.62	73.26	6.72	7.79	6.59	4.70	0.94
Myiopsitta monachus	0.65	82.71	16.64	65.40	8.53	7.96	12.31	4.63	1.17
Amazona aestiva	0.55	83.46	15.99	69.58	8.79	6.83	10.33	3.62	0.85
Melopsittacus undulatus	0.46	84.61	14.93	61.26	10.40	7.79	14.85	3.82	1.88
Rhegmatorhina hoffmannsi	0.64	84.50	14.86	49.65	14.18	11.59	15.24	6.90	2.44
Certhia americana	1.19	77.20	21.62	65.43	9.94	7.79	9.96	4.97	1.90
Hirundo rustica	1.70	73.97	24.34	65.26	7.80	7.46	11.44	6.22	1.82
Catharus ustulatus	1.03	81.54	17.43	53.58	8.44	10.11	15.69	8.73	3.45
Erithacus rubecula	1.39	77.04	21.57	59.77	8.30	7.32	13.86	8.09	2.66

Ficedula albicollis	2.24	74.77	22.99	67.60	6.65	5.87	11.98	5.11	2.79
Sylvia atricapilla	1.29	77.52	21.18	65.93	8.34	8.38	9.36	6.35	1.65
Sylvia borin	1.21	78.78	20.02	64.08	8.58	9.43	10.10	5.76	2.05
Acrocephalus scirpaceus	0.94	81.41	17.65	57.79	9.58	10.58	12.66	7.23	2.16
Parus major	0.70	82.86	16.44	56.11	13.33	11.28	10.90	5.76	2.62
Camarhynchus parvulus	1.14	82.12	16.75	46.41	11.32	12.45	16.11	10.85	2.86
Diglossa brunneiventris	1.14	80.36	18.50	54.76	10.49	9.89	14.15	8.69	2.02
Passer domesticus	0.55	83.57	15.88	60.78	9.94	10.02	11.64	5.94	1.68
Molothrus ater	0.95	81.21	17.84	52.31	12.34	12.00	13.72	7.43	2.20
Setophaga coronata	0.51	84.60	14.89	58.02	12.67	10.91	11.59	5.57	1.23
Geothlypis trichas	1.14	81.75	17.12	49.39	12.87	11.04	15.43	9.34	1.94
Motacilla alba	1.01	80.03	18.97	59.78	10.42	9.42	12.39	6.02	1.96
Fringilla coelebs	0.54	83.19	16.27	62.08	9.74	10.42	10.79	5.27	1.70
Lonchura striata	1.45	79.00	19.54	64.22	9.03	8.60	9.53	5.82	2.80
Taeniopygia guttata	1.22	78.21	20.57	62.72	7.98	7.98	10.37	8.35	2.60
Corvus moneduloides	0.80	81.83	17.37	58.33	10.11	9.80	12.98	6.16	2.64
Corvus monedula	0.86	81.46	17.67	59.76	9.35	9.14	13.20	6.87	1.69
Malurus cyaneus	1.30	78.93	19.78	66.64	6.96	6.71	10.38	7.55	1.76
Streptopelia turtur	0.89	78.98	20.13	64.92	8.87	6.53	12.72	5.84	1.12
Phoenicopterus ruber	0.54	80.72	18.74	72.68	6.30	8.21	7.39	4.54	0.87
Ciconia maguari	0.56	81.67	17.77	68.69	7.74	9.33	7.48	5.44	1.32
Gymnogyps californianus	0.51	80.49	19.00	70.37	6.37	10.93	7.17	4.23	0.93
Alca torda	0.80	77.91	21.29	76.17	6.55	5.47	6.99	4.04	0.78
Sterna hirundo	0.72	78.69	20.59	75.38	6.72	6.48	6.86	3.73	0.82
Pluvialis apricaria	0.64	80.10	19.27	73.85	6.62	7.23	6.68	4.41	1.23
Falco naumanni	0.81	79.79	19.40	72.92	8.50	6.90	7.65	3.22	0.81

Falco rusticolus	0.69	80.63	18.69	72.15	8.97	7.22	7.64	3.30	0.72
Aquila chrysaetos	0.65	78.09	21.26	76.48	6.30	7.39	6.21	3.07	0.56
Calypte anna	1.02	79.15	19.84	64.51	9.01	7.07	10.58	6.88	1.94
Caprimulgus europaeus	0.80	80.70	18.50	63.52	10.60	8.56	9.95	5.68	1.69
Nyctibius grandis	0.39	83.97	15.65	71.80	7.97	8.39	6.58	4.03	1.23

Table S4. The top five most abundant repeat motif of P-SSRs.

Species	1st	%	2nd	%	3rd	%	4th	%	5th	%
Coturnix japonica	A	46.64	AAAT	6.69	AT	6.32	AAAC	5.78	C	4.33
Meleagris gallopavo	A	57.98	AAAC	5.87	AT	4.90	AAAT	4.64	AC	4.09
Gallus gallus	A	54.12	C	7.22	AAAC	5.20	AT	3.99	AAAT	3.28
Numida meleagris	A	68.22	C	3.66	AT	3.45	AAAC	2.83	AC	2.70
Oxyura jamaicensis	A	57.19	C	5.43	AAAT	5.42	AT	5.17	AAAC	5.07
Heteronetta atricapilla	A	58.45	AT	5.55	AAAT	5.10	AAT	4.89	AAAC	4.82
Cygnus olor	A	55.18	C	6.03	AT	4.56	AAAT	4.53	AAAC	4.39
Aythya fuligula	A	59.42	AAAC	5.51	AAAT	5.39	AT	4.29	AAT	3.36
Anas platyrhynchos	A	56.26	AAAT	5.85	AAAC	5.44	AT	4.57	C	3.44
Stictonetta naevosa	A	57.86	AAAT	5.51	AAAC	5.36	C	4.65	AAT	4.40
Cairina moschata	A	61.02	AAAT	5.73	AAAC	5.18	AT	4.72	AAT	3.39
Colaptes auratus	A	25.42	C	7.23	AATAG	6.82	AAGAT	6.61	AATAC	6.11
Pogoniulus pusillus	A	36.21	C	11.26	AC	5.49	AT	5.08	AG	4.21
Bucorvus abyssinicus	A	68.07	C	5.19	AC	3.05	AT	2.32	AGG	1.83
Myiopsitta monachus	A	52.36	C	13.04	AC	3.85	AT	3.24	AAAC	2.82
Amazona aestiva	A	66.74	AAAC	4.21	AT	4.08	AC	3.84	C	2.84
Melopsittacus undulatus	A	44.13	C	17.13	AT	5.18	AAAG	5.13	AC	4.36
Rhegmatorhina hoffmannsi	A	44.19	AC	7.14	C	5.46	AT	4.62	AAAC	4.24
Certhia americana	A	61.52	AT	4.24	AC	4.12	C	3.91	AAAC	2.68
Hirundo rustica	A	62.06	AAAC	5.12	AC	3.22	C	3.21	AT	3.01
Catharus ustulatus	A	51.43	AC	4.07	AT	3.10	AAAC	2.73	AAT	2.30
Erithacus rubecula	A	56.93	AAGG	4.03	AT	3.38	AC	3.34	C	2.84
Ficedula albicollis	A	45.15	C	22.45	ATCC	4.44	AC	2.83	AT	2.65

Sylvia atricapilla	A	63.82	AT	3.59	AAAC	3.41	AC	3.08	C	2.10
Sylvia borin	A	61.9	AAAC	3.81	AT	3.53	AC	3.17	C	2.18
Acrocephalus scirpaceus	A	55.24	AT	4.11	AC	3.55	AAAC	3.00	AAT	2.64
Parus major	A	55.09	AT	6.76	AC	4.74	AAT	3.23	AAAC	2.58
Camarhynchus parvulus	A	44.68	AC	4.85	AT	4.63	AAAC	2.95	AGG	2.82
Diglossa brunneiventris	A	52.19	AC	4.6	AT	4.15	ATCC	3.18	C	2.57
Passer domesticus	A	60.05	AC	4.29	AT	3.99	AAAC	3.42	AAT	2.21
Molothrus ater	A	48.98	AC	5.11	AT	4.91	ATCC	3.56	C	3.33
Setophaga coronata	A	57.54	AT	6.19	AC	4.66	AAT	3.13	AAAC	3.06
Geothlypis trichas	A	46.65	AT	5.88	AC	4.84	AAGG	2.84	C	2.73
Motacilla alba	A	56.84	AT	4.62	AC	4.10	C	2.95	AAAC	2.80
Fringilla coelebs	A	60.55	AC	4.17	AT	4.03	AAAC	2.61	AAT	2.31
Lonchura striata	A	62.42	AT	4.42	AC	3.36	AAAC	2.81	AGG	2.18
Taeniopygia guttata	A	59.91	AT	3.65	AC	3.12	C	2.81	ATCC	2.47
Corvus moneduloides	A	52.95	C	5.38	AC	4.15	AT	4.01	AAGG	3.33
Corvus monedula	A	55.14	C	4.62	AT	3.98	AC	3.65	AAGG	3.52
Malurus cyaneus	A	64.55	AC	2.97	AT	2.60	C	2.09	AAT	1.72
Streptopelia turtur	A	62.58	AAAC	6.39	AT	5.02	AC	3.00	C	2.34
Phoenicopterus ruber	A	62.78	C	9.91	AC	2.86	AAT	2.31	AAAC	2.02
Ciconia maguari	A	57.66	C	11.03	AC	3.29	AAT	2.97	AT	2.52
Gymnogyps californianus	A	59.53	C	10.84	AAT	4.05	AC	3.30	AGG	2.16
Alca torda	A	67.67	C	8.5	AC	2.85	AT	2.40	AAAC	1.68
Sterna hirundo	A	65.71	C	9.68	AC	3.17	AT	2.10	AAAC	1.66
Pluvialis apricaria	A	61.72	C	12.13	AC	3.63	AGG	1.76	AAAC	1.68
Falco naumanni	A	60.06	C	12.86	AC	4.04	AT	3.34	AAAC	2.03
Falco rusticolus	A	60.23	C	11.93	AC	4.24	AT	3.55	AAAC	2.06

Aquila chrysaetos	A	61.41	C	15.07	AC	2.90	AT	2.17	AAAC	1.85
Calypte anna	A	61.41	AT	3.98	AC	3.47	AAAC	3.18	C	3.10
Caprimulgus europaeus	A	59.72	AT	4.85	AC	4.29	C	3.80	AAAC	2.93
Nyctibius grandis	A	67.11	C	4.69	AC	3.41	AAT	2.83	AT	2.78

Table S5. The most abundant repeat motif of six P-SSR types.

Species	Mono-	%	Di-	%	Tri-	%	Tetra-	%	Penta-	%	Hexa-	%
Coturnix japonica	A	46.64	AT	6.32	AAT	4.01	AAAT	6.69	AAAAC	1.41	AAAAAG	0.40
Meleagris gallopavo	A	57.98	AT	4.90	AAT	2.87	AAAC	5.87	AAAAC	1.57	AATCCC	0.27
Gallus gallus	A	54.12	AT	3.99	AAT	2.37	AAAC	5.20	AAAAC	1.13	AACCCT	0.84
Numida meleagris	A	68.22	AT	3.45	AAT	1.86	AAAC	2.83	AAAAC	0.78	AAAAAG	0.13
Oxyura jamaicensis	A	57.19	AT	5.17	AAT	4.85	AAAT	5.42	AAAAC	1.53	AAAAAG	0.18
Heteronetta atricapilla	A	58.45	AT	5.55	AAT	4.89	AAAT	5.10	AAAAC	1.25	AAAAAG	0.17
Cygnus olor	A	55.18	AT	4.56	AAT	3.53	AAAT	4.53	AAAAC	1.27	AAAAAG	0.51
Aythya fuligula	A	59.42	AT	4.29	AAT	3.36	AAAC	5.51	AAAAC	1.76	AATCCC	0.28
Anas platyrhynchos	A	56.26	AT	4.57	AAT	3.42	AAAT	5.85	AAAAT	1.82	AATCCC	0.93
Stictonetta naevosa	A	57.86	AT	4.34	AAT	4.40	AAAT	5.51	AAAAC	1.60	AAAAAG	0.21
Cairina moschata	A	61.02	AT	4.72	AAT	3.39	AAAT	5.73	AAAAT	1.61	AAAAAT	0.17
Colaptes auratus	A	25.42	AC	3.99	AAT	1.46	AAAC	2.97	AATAG	6.82	AAAAAG	0.24
Pogoniulus pusillus	A	36.21	AC	5.49	ACG	3.56	AAAC	3.42	AGAGG	0.84	AAAAAG	0.30
Bucorvus abyssinicus	A	68.07	AC	3.05	AGG	1.83	AAAC	1.74	AAGGG	0.83	AAAAAG	0.09
Myiopsitta monachus	A	52.36	AC	3.85	AAT	2.06	AAAC	2.82	AAAAC	0.82	AACCCT	0.15
Amazona aestiva	A	66.74	AT	4.08	AAT	2.10	AAAC	4.21	AAAAC	1.41	AACCCT	0.14
Melopsittacus undulatus	A	44.13	AT	5.18	AAT	2.41	AAAG	5.13	ATCCC	0.60	AATCCC	0.92
Rhegmatorhina hoffmannsi	A	44.19	AC	19.83	AGG	19.31	AAAC	4.24	AAAAC	1.00	ACAGGG	0.22
Certhia americana	A	61.52	AT	4.24	AAT	1.99	AAAC	2.68	AAAAC	0.74	ACAGGG	0.16
Hirundo rustica	A	62.06	AC	3.22	AAC	2.26	AAAC	5.12	AAAAC	1.45	ACAGGG	0.18
Catharus ustulatus	A	51.43	AC	4.07	AAT	2.30	AAAC	2.73	ATCCC	1.22	ACAGGG	0.43
Erithacus rubecula	A	56.93	AT	3.38	AAT	1.55	AAGG	4.03	AGAGG	1.22	ACAGGG	0.26
Ficedula albicollis	A	45.15	AC	2.83	AAT	1.40	ATCC	4.44	AAAAC	0.80	AATCCC	0.23

Sylvia atricapilla	A	63.82	AT	3.59	AAT	1.98	AAAC	3.41	AAAAC	1.19	AAAAAG	0.18
Sylvia borin	A	61.90	AT	3.53	AAT	2.10	AAAC	3.81	AAAAC	1.26	ACAGGG	0.24
Acrocephalus scirpaceus	A	55.24	AT	4.11	AAT	2.64	AAAC	3.00	AAAAT	0.83	AAAAAG	0.25
Parus major	A	55.09	AT	6.76	AAT	3.23	AAAC	2.58	AAAAT	0.95	AAATAT	0.23
Camarhynchus parvulus	A	44.68	AC	4.85	AGG	2.82	AAAC	2.95	AATAT	1.29	ACAGGG	0.23
Diglossa brunneiventris	A	52.19	AC	4.60	AAT	2.17	ATCC	3.18	AATAT	1.24	AAAAAG	0.17
Passer domesticus	A	60.05	AC	4.29	AAT	2.21	AAAC	3.42	AAAAC	1.05	AAAAAG	0.16
Molothrus ater	A	48.98	AC	5.11	AGG	2.64	ATCC	3.56	ATCCC	0.93	ACAGGG	0.19
Setophaga coronata	A	57.54	AT	6.19	AAT	3.13	AAAC	3.06	AATAT	0.83	AAATAT	0.12
Geothlypis trichas	A	46.65	AT	5.88	AAT	2.59	AAGG	2.84	AATAT	1.38	AAATAT	0.15
Motacilla alba	A	56.84	AT	4.62	AAT	2.03	AAAC	2.80	AAAAC	0.85	AAAAAG	0.16
Fringilla coelebs	A	60.55	AC	4.17	AAT	2.31	AAAC	2.61	AAAAC	0.68	ATATAG	0.14
Lonchura striata	A	62.42	AT	4.42	AGG	2.18	AAAC	2.81	AAAAC	0.98	ATATAG	0.21
Taeniopygia guttata	A	59.91	AT	3.65	AGG	1.96	ATCC	2.47	AAGGG	1.55	ACAGGG	0.24
Corvus moneduloides	A	52.95	AC	4.15	AGG	2.59	AAGG	3.33	ATCCC	0.83	AATCCC	0.89
Corvus monedula	A	55.14	AT	3.98	AAT	2.10	AAGG	3.52	AAGGG	1.01	AAAAAG	0.23
Malurus cyaneus	A	64.55	AC	2.97	AAT	1.72	AAAC	1.64	AGAGG	1.32	ACAGGG	0.19
Streptopelia turtur	A	62.58	AT	5.02	AAC	1.98	AAAC	6.39	AAAAC	1.44	AAAAAG	0.13
Phoenicopterus ruber	A	62.78	AC	2.86	AAT	2.31	AAAC	2.02	AAAAC	0.58	CCCCGG	0.14
Ciconia maguari	A	57.66	AC	3.29	AAT	2.97	AAAC	2.10	AAAAC	0.60	CCCCGG	0.22
Gymnogyps californianus	A	59.53	AC	3.30	AAT	4.05	AAAC	1.91	AAAAC	0.45	CCCCGG	0.13
Alca torda	A	67.67	AC	2.85	AGG	1.41	AAAC	1.68	AAAAC	0.65	AAAAAG	0.13
Sterna hirundo	A	65.71	AC	3.17	AGG	1.62	AAAC	1.66	AAAAC	0.57	AAAAAG	0.16
Pluvialis apricaria	A	61.72	AC	3.63	AGG	1.76	AAAC	1.68	AAAAC	0.54	AAAAAG	0.15
Falco naumanni	A	60.06	AC	4.04	AAT	1.77	AAAC	2.03	AAAAC	0.50	AATCCC	0.13
Falco rusticolus	A	60.23	AC	4.24	AAT	1.81	AAAC	2.06	AAAAC	0.52	AAAAAG	0.07

Aquila chrysaetos	A	61.41	AC	2.90	AAT	1.71	AAAC	1.85	AAAAC	0.52	CCCCGG	0.11
Calypte anna	A	61.41	AT	3.98	AAT	1.96	AAAC	3.18	AAAAC	1.05	AAAAAG	0.32
Caprimulgus europaeus	A	59.72	AT	4.85	AAT	2.75	AAAC	2.93	AAAAT	0.91	AAAAAG	0.31
Nyctibius grandis	A	67.11	AC	3.41	AAT	2.83	AAAC	1.79	AAAAC	0.61	AAAAAG	0.21
